# Genetic Risk in Coronary Artery Disease

**DOI:** 10.5935/abc.20180130

**Published:** 2018-07

**Authors:** Paula F. Martinez, Marina P. Okoshi

**Affiliations:** 1 Faculdade de Fisioterapia da Universidade de Mato Grosso do Sul, Campo Grande, MS - Brazil; 2 Faculdade de Medicina de Botucatu (UNESP), Botucatu, SP – Brazil

**Keywords:** Coronary Artery Disease/genetics, Polymorphism, Genetic, Genome-Wide Association Study

Coronary artery disease (CAD) is a leading cause of death worldwide. It is most commonly caused by atherosclerosis in coronary arteries. Coronary artery disease has a complex etiology, mainly a combination of traditional risk factors and genetic predisposition. Traditional risk factors include type 2 diabetes, dyslipidemia, arterial hypertension, and cigarette smoking.^1^ However, these are not sufficient to identify high risk asymptomatic individuals and do not explain all cases of CAD. In fact, hereditary influence on CAD susceptibility accounts for between 40% and 50% of cases.^2^

Polymorphisms are common genetic variations, defined as being present in more than 1% of the population.^3^ A polymorphism is a nucleotide substitution that does not alter the primary amino acid structure of the resulting protein.^3^ A single-nucleotide polymorphism (SNP) is a variation in DNA in a single nucleotide that occurs at a specific position in the genome. An SNP may be a marker of disease susceptibility.^3^ Populations of healthy and affected individuals can be evaluated by genotyping SNP within a gene and its regulatory sequences.^4^ Genome-wide association studies (GWAS) have been used to create genetic risk scores to improve CAD risk prediction.^4-6^ However, their value as an independent risk predictor for CAD is not clear.

In this issue of *Arquivos Brasileiros de Cardiologia*, Pereira et al.^7^ provide us with an interesting study on generating a multilocus genetic risk score based on common variants already associated with CAD. They then evaluated whether genetic risk score is independent of the traditional risk factors and improves CAD risk prediction in relation to a traditional risk factor only model.

By searching data from the National Human Genome Research Institute, the authors analyzed 33 genetic variants previously associated with CAD. The study population was selected from GENEMACOR (*GENEs in a population from the Portuguese island of MAdeira with CORonary artery disease*), a developing case-control population study with 1,566 cases and 1,322 controls. Coronary risk was determined by logistic regression analysis. Two ROC curves were constructed, one with and one without genetic risk score; these were compared by use of the DeLong test. The estimated area under the traditional risk factor ROC curve was 0.72, which statistically increased to 0.74 when the genetic risk score was added, thus revealing a better fit of the model. The study strength comes from assessing a large sample size and a homogenous population as only permanent Madeira residents were included.

Genetic risk scores have undergone extensive study and major progress has been made to better understand the role of genetic influence on CAD and the function of each novel locus.^4,8-13^ However, the role of most genetic variants in disease processes remains unknown.^10^ Furthermore, the presence or lack of a traditional risk factor may determine whether or not a genetic factor will contribute to disease.^5^

Although in the study by Pereira et al.^7^ the addition of genetic risk score gave a statistically superior score in identifying high risk patients, the difference between the two risk factor curves was small. Therefore, considering that traditional risk factors have been poorly controlled in the general population and the high financial cost of determining genetic risk scores, it is important to remain focused on preventing and controlling traditional risk factors until the role of genetic risk scores is better understood.
